# Stigma, Discrimination and Disclosure of the Diagnosis of Multiple Sclerosis in the Workplace: A Systematic Review

**DOI:** 10.3390/ijerph19159452

**Published:** 2022-08-02

**Authors:** Bruno Kusznir Vitturi, Alborz Rahmani, Guglielmo Dini, Alfredo Montecucco, Nicoletta Debarbieri, Paolo Bandiera, Michela Ponzio, Mario Alberto Battaglia, Benedetta Persechino, Matilde Inglese, Paolo Durando

**Affiliations:** 1 Department of Health Sciences, University of Genoa, 16132 Genoa, Italy; alborz.rahmani@edu.unige.it (A.R.); guglielmo.dini@unige.it (G.D.); alfredo.montecucco@edu.unige.it (A.M.); paolo.durando@unige.it (P.D.); 2 Occupational Medicine Unit, IRCCS Ospedale Policlinico San Martino, 16132 Genoa, Italy; nicoletta.debarbieri@hsanmartino.it; 3 Italian Multiple Sclerosis Association (AISM), 16126 Genoa, Italy; paolo.bandiera@aism.it; 4 Scientific Research Area, Italian Multiple Sclerosis Foundation (FISM), 16126 Genoa, Italy; michela.ponzio@aism.it (M.P.); m.a.battaglia@aism.it (M.A.B.); 5 Department of Life Science, University of Siena, 53100 Siena, Italy; 6 Italian Workers’ Compensation Authority (INAIL), 00078 Rome, Italy; b.persechino@inail.it; 7 Department of Neurosciences, Rehabilitation, Ophthalmology, Genetics, Maternal and Child Health (DiNOGMI), University of Genoa, 16132 Genoa, Italy; m.inglese@unige.it; 8 Neurology Unit, IRCCS Ospedale Policlinico San Martino, 16132 Genoa, Italy

**Keywords:** multiple sclerosis, demyelinating disease, work, occupational medicine, stigma, discrimination, job, neurology

## Abstract

The objective of the study was to describe and analyze the stigma, discrimination and the disclosure of the diagnosis of Multiple Sclerosis (MS) in the workplace. The protocol was registered in PROSPERO (CRD42022320437). We systematically searched four scientific databases with key search terms. We included any original peer-reviewed articles reporting the stigma or discrimination experienced at work due to MS or the disclosure of the diagnosis of MS in the workplace. No time limits were set for the search. An appraisal of the individual study quality was performed with the JBI critical appraisal checklist. Overall, 26 studies were deemed to fulfil all the eligibility criteria. The total number of participants in this review was 9571. The prevalence of people with MS who experience some degree of stigma in the workplace can be as high as 79.2%. Those who report greater feelings of discrimination are more likely to be unemployed. The prevalence of employers’ and co-workers’ awareness of the diagnosis varies from 31.7 to 90.2%. The main reason for non-disclosure is the fear of being discriminated against. The psychosocial work environment needs to be taken into consideration as part of public and individual policies to promote the health of patients with MS.

## 1. Introduction

Multiple Sclerosis (MS) is a chronic autoimmune disease that causes demyelination and neurodegeneration in the central nervous system. It mainly affects young people between 20 and 40 years of age and it is the main cause of non-traumatic disability among young adults in the western world [1]. MS is a global disease whose incidence and prevalence are known to be increasing in both developed and developing countries [2]. The symptoms are extremely varied and the clinical course extends from relapsing to progressive [1].

In addition to the inherent clinical complexity of MS, the age of onset of the disease brings inevitable repercussions to work activity, as it often coincides with the time in patients’ lives when they find themselves managing the already expected difficulties of the job market and the beginning of a professional career. Due to the variety of symptoms and the epidemiology of the disease, MS is one of the most challenging neurological diseases in an occupational context [3]. No more than 17% of people with MS (PwMS) are spared from any kind of problem at work due to the illness [4]. Some of the consequences of MS at work can be invisible or neglected such as the stigma and discrimination experienced by PwMS. Stigma and discrimination in the workplace are well-known psychosocial stressors that are still typically unpredictable and uncontrollable in the occupational context. Employees suffering from adverse psychosocial circumstances at work are more vulnerable to stress, and low self-esteem and may leave their jobs prematurely as a means of coping [5]. The perception of stigma and discrimination is associated with lower quality of life and greater difficulties at work [6]. In addition, negative interpersonal relationships in the workplace may be associated with the onset of other diseases [7,8,9,10].

The psychosocial context of the work environment is directly associated with the disclosure of the diagnosis of MS. For many PwMS, disclosure of their diagnosis at work is seen as a high-risk strategy that might lead to diminished perceptions of their capabilities by supervisors and colleagues, if not outright discrimination. In some cases, the decision may be inevitable and PwMS may be required to disclose the diagnosis because of the severity of the disease, for example. The value of talking about the illness has been recognized as playing an important role in helping people to work through their difficulties in some cases [11]. The non-disclosure of the diagnosis directly interferes with the need for job accommodations and prevents the prompt identification of the work barriers [12]. In addition, PwMS may not even report the diagnosis to the occupational physician, who thus has a limited role in preventing unfavourable occupational outcomes [13].

There is a growing interest in characterizing illness-related stigma and discrimination in the workplace and evidence-based best practices to overcome them. In parallel, the dissemination of the diagnosis of MS can be understood as a sentinel of the integration of the worker into the workplace, which is essential for job retention. Nevertheless, no article systematically summarises the information already published on the subject. An evidence-based characterization of stigma, discrimination, and disclosure of the diagnosis of MS is fundamental to promoting the quality of life of PwMS and optimal occupational outcomes. The primary objectives of the present systematic review are to examine the characteristics and impact of stigma, discrimination, and disclosure of the diagnosis of MS at work. The secondary aims are to report the prevalence of these outcomes and the clinical, demographic, and occupational factors associated with them.

## 2. Materials and Methods

This study was carried out according to the Preferred Reporting Items for Systematic Reviews and Meta-Analyses Statement (PRISMA). The protocol was registered in PROSPERO (CRD42022320437). As this was a literature review, it did not involve the recruitment of subjects and it analyzed data from already published original articles; therefore, the ethical approval was not necessary. 

From 1 August 2021, to 30 October 2021, we systematically searched on PubMed/MEDLINE, Scopus, SciVerse ScienceDirect, and Web of Science the following keywords (Employ* OR unemploy* OR occupation* OR “work” OR vocation* OR “work resumption” OR workplace* OR “return to work” OR “workforce” OR “workforce” OR “labour force” OR “labor force” OR Career* OR Job* OR “job retention” OR retire* OR “disability pension” OR “worker” OR “fitness for work”) AND (“Multiple sclerosis” OR “Disseminated Sclerosis” OR “Demyelinating Autoimmune Diseases” OR “Demyelinating Autoimmune Disorders” OR “Clinically Isolated Syndrome” OR “Demyelinating”). The details of the search strategy used are reported in Table 1. We did not explore any grey literature sources. We adopted a broad search methodology to ensure the maximum inclusion of studies reporting both outcomes. After the preliminary identification, the articles were exported and managed in Mendeley 1.19.8 (Elsevier, New York, NY, USA). 

Articles were selected according to the PICo (Population/Interest/Context) strategy. We included any original peer-reviewed articles reporting the stigma or discrimination experienced at work due to MS or the disclosure of the diagnosis of MS in the workplace. MS must have been diagnosed according to accepted international criteria at the time of the study or confirmed by a doctor. No time limits were set for the search. We accepted articles that were published in English, Italian, Spanish, French, and Portuguese. Data were taken from cross-sectional studies and baseline measurements of longitudinal and interventional studies.

After we removed duplicate entries, we performed an initial screening of titles or abstracts to assess potential relevance and remove those off-topic. Each article was screened by three experienced and trained investigators (BKV, AR, and AM), each blinded to the other’s ratings. In the case of discrepancy, a final decision was made by consensus. Afterward, we obtained relevant full-text articles, revaluated their eligibility, and determined their final inclusion or exclusion. 

Studies written in languages other than the five pre-specified above and studies designed as reviews, letters to the editor, expert opinions, commentaries, case reports, case series, and editorials were excluded. In the case of articles with missing or dubious data or without an available full text, we tried to contact the corresponding author twice to obtain more information by email. The study was excluded whenever our contact attempt failed. We didn’t accept studies whose sample deliberately included patients with more than a chronic disease or in which MS was not the primary condition. When multiple articles reported data from the same population, the article with the highest number of variables described was selected. 

Data extraction was also performed by two independent reviewers (BKV and AR) and eventual disagreements were resolved by discussion until a consensus was reached. Data on the first author, year of publication, country, sample size, mean age, gender, higher educational attainment (defined as >12 schooling years), study design, mean disease duration, MS phenotype (progressive or relapsing-remitting) were extracted and tabulated in a Microsoft Excel spreadsheet. Not only the description and characterization of the outcomes were extracted from the studies but also eventual data associated with the context in which they were investigated. The main characteristics and results of the studies were synthesized in a table. It was not possible to carry out a meta-analysis due to the diversity of variables and definitions found in the articles and a lack of quantitative information in some cases. Therefore, a narrative synthesis of the key findings was performed. 

An appraisal of individual study quality was performed with the JBI critical appraisal checklist (for cross-sectional, cohort, qualitative, quasi-experimental, and experimental studies). Each checklist contains 8 to 13 questions for which trained reviewers can select “yes”, “no”, “uncertain”, or “not applicable (NA)” in response to each item. Whenever “no” or “uncertain” has been selected, it should be interpreted as a potential flaw. Each article was rated independently by two reviewers (BKV and GD). If the ratings differed, the reviewers discussed the article to reach a consensus.

## 3. Results

**Figure 1:**
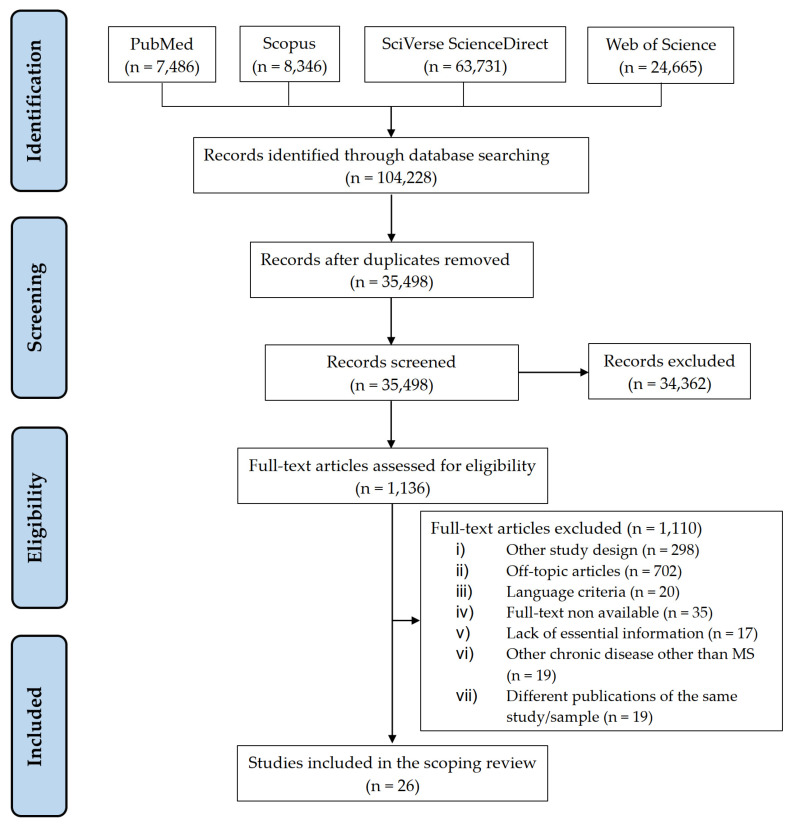
PRISMA flowchart.

The initial database search yielded 104,228 articles. Of these, 68,730 were duplicates. After applying the exclusion criteria, a total of 26 studies were deemed to fulfil all eligibility criteria and were thus included in the review (Figure 1). The 26 articles included in the present review were published between 1993 and 2021 (Table 2). Most of them have a high methodological quality—the detailed critical appraisal of the studies is described in Table 3. Overall, the studies were conducted in ten countries: The United States of America (9, 34.7%), Australia (4, 15.5%), France (3, 11.6%), Spain (2, 7.7%), Iran (2, 7.7%), Canada (1, 3.8%), Ireland (1, 3.8%), New Zealand (1, 3.8%), Poland (1, 3.8%), and the United Kingdom (1, 3.8%). One (3.8%) study was performed in multiple countries. Sixteen (61.6%) were cross-sectional studies, 4 (15.4%) were cohort studies, 3 (11.5%) were qualitative studies, 2 (7.7%) were quasi-experimental studies and 1 (3.8%) was an experimental study. The total number of participants in this review was 9571 (range: 6–1924 per study). The mean age ranged from 31.2 to 54.0 years while the mean disease duration ranged from 6.4 to 18.9 years. The proportion of women varied from 18.9% to 93.1%. Twelve (46.2%) studies reported data on stigma and/or discrimination, 9 (34.6%) on disclosure of the diagnosis in the workplace and 5 (19.2%) addressed both topics. No study addressed how the MS heterogeneity may be associated with the stigma, discrimination and the disclosure of the diagnosis of MS. Typically, the only MS variables reported were disease duration and clinical phenotype, but there was no study looking at their particular influence on the outcomes. 

PwMS are vulnerable to stigma at work and some evidence suggests that those who have a non-governmental job or are unemployed perceive it more often [6,26]. The prevalence of PwMS who experience some degree of stigma can be as high as 79.2% [31]. Workers with progressive MS and greater disability are at risk of experiencing stigma in the workplace [6]. PwMS that report greater feelings of stigmatization are more likely to be experienced by the unemployed (OR = 7.42, 95% CI 2.59–21.28), people with a poorer quality of life (OR = 13.12, 95% CI 5.51–31.20), and people requiring informal care (OR = 3.83, 95% CI 1.84–7.96). Moreover, feelings of stigmatization are directly associated with depression, cognitive impairment, and disability [23]. A French study found that fear of stigmatization can prevent workers from requesting reasonable workplace adjustments [19]. Similarly, Gill et al. showed that PwMS may not be comfortable bringing supportive aids into the workplace, believing that their colleagues would see them “differently” [21]. Honan et al. found that PwMS consider low self-esteem and lack of support from co-workers to be very important work barriers [24]. Unfortunately, no studies have sought to objectively specify discriminatory acts perceived by PwMS.

Some workers consider the invisibility of symptoms as a challenge to an accurate interpretation of the disease, leading to mistaken and biased opinions about their illness [21,32]. Most people with MS consider the lack of knowledge about MS as a key element of discriminatory attitudes [35]. Indeed, after the disclosure of the diagnosis, only 24.1%, 26.7%, and 32.5% of PwMS believe that the director of human resources, colleagues, and managers, respectively, had sufficient knowledge about their illness [19]. 

Dorstyn et al. developed a job-information resource aimed at changing the psychosocial working conditions. The strategy would promote a positive job identity and general mental health in the work environment. The researchers found that workers who had access to the program improved expectations in relation to the effect of MS symptoms on general self-esteem (*p* = 0.02) and work social relations (*p* = 0.03) [18]. Sweetland et al. showed that workers with MS wished to receive information about discrimination and its management [35]. In line with these findings, Rumrill et al. found that 96.5% would like to know how to react to discrimination at work [34]. 

The prevalence of employers’ and co-workers’ awareness of the diagnosis of MS varies from 31.7 to 90.2% [12,13,15,16,20,22,25,27,29,30,36]. This prevalence remains stable and does not change significantly over a 3-year period [12]. Not only may PwMS fail to report their diagnosis to employers and colleagues, but also to occupational physicians. Abbas et al. reported that 42% of the occupational physicians were unaware of the existence of MS [13]. Ongagna et al. report a prevalence of 81.6% of patients declaring their diagnosis to the occupational physician [30]. In a cross-sectional study with 941 participants, 89.5% of workers disclosed MS to their occupational physicians at some point. The respondents also stated that they believed that 80.6% of occupational physicians had satisfactory knowledge of their illness [19].

The issue of disclosure of diagnosis in the workplace concerns PwMS both when they are seeking employment and when they are already employed. In the first scenario, PwMS think that by announcing their illness, employers would automatically be more inclined to choose other candidates [26,28]. In the second situation, the decision to conceal their illness in the working environment is mostly explained by the fear of losing their jobs [13] or creating work conflicts [17]. More than 97% of PwMS weigh up the risks and benefits of disclosing the neurological disease to the employer [33]. Frndak et al. also showed that workers with MS feel more uncomfortable communicating the diagnosis when they have a continued positive performance and a new-hire status [20]. Moreover, MS symptoms may force the worker to disclose his illness. Indeed, Bass et al. suggest that as some symptoms are invisible, PwMS may be more likely to conceal their diagnosis despite significant health distress and impairment of social relationships [15]. 

Dorstyn et al. show that PwMS fear being discriminated against after disclosing their neurological disease [17]. Indeed, Fantoni-Quinton reported that the fear of being stigmatized and side-lined could explain the reticence to disclose the diagnosis in some cases [19]. Nevertheless, Gregory et al. report that only 7% of employers were not sympathetic to the employee and his or her neurological condition [22]. Similarly, disclosure was associated with a lower probability of negative employer attitudes (OR = 0.60, 95% CI 0.48–0.74) [12]. PwMS reveal they would be more empowered to disclose their illness at work if they better understood how they were protected legally [35]. A good relationship with the employer may facilitate the process of disclosing the diagnosis [20]. 

Benedict et al. found that cognitive impairment, psychiatric symptoms, educational level, and age are not associated with the disclosure of MS [16]. These findings were complemented by a successive study that demonstrated that disclosure sub-groups did not differ in age, gender, educational level, monthly income, or cognitive performance. In contrast, disclosure was associated with having worked longer for the current employer (*p* = 0.007), working more hours per week (*p* = 0.036), and using more accommodations (*p* = 0.001). In addition, people who disclosed were found to have more advanced disease (*p* = 0.022) and greater disabilities (*p* = 0.022) [20]. Kirk-Brown et al. also found that workers with a more severe disability are more likely to disclose the diagnosis [12]. 

The association between diagnosis disclosure and employment status remains controversial in the literature. One qualitative study presents reports of workers who attribute their dismissal from employment to the employer’s knowledge of the diagnosis [32]. Some workers with MS judge the decision to disclose the illness as a key element to preventing unemployment, considering that the unpredictability of symptoms may impact others in the work environment [21]. A French study showed that the prevalence of workers who disclose the diagnosis was not statistically significant between those who lost their jobs and those who were still employed [13]. Notwithstanding, an Australian cohort study found that the odds of a person with MS remaining in employment increased by 1.30 (95% CI 1.07–1.57) when the employee disclosed the diagnosis to the manager. The disclosure also represented a 3.35-year increase in job tenure [12]. Studies describe that PwMS who had announced their illness in their work environments complained of misunderstandings and lack of support from others [14]. 

## 4. Discussion

This review demonstrated that a large proportion of patients with MS face stigma and discrimination in the workplace and many prefer not to disclose their illness to their colleagues. Patients with more severe diseases are more likely to be stigmatized and more likely to communicate their diagnosis. It is reasonable to suppose that many work-related factors may account for the decision to disclose and the perception of stigma in the workplace due to MS. Nevertheless, there is a lack of evidence linking these outcomes to the work characteristics and job types. Likewise, there are no studies that evaluate the stigma and discrimination directly from the employer and co-workers, as well as their reactions to the disclosure of the diagnosis. Moreover, no study assessed the impact of stigma and discrimination on the MS symptoms, albeit previous studies have already revealed the possible influence of work psychosocial characteristics on the disease itself [5]. Indeed, the work context plays a critical role in the sustainable employment of people with disabilities or neuropsychiatric conditions [37]. 

Stigma, discrimination, and disclosure of the diagnosis of MS at work are interrelated and should be addressed together [5]. MS-related stigma often leads to discriminatory behavior by employers. Patients with MS who feel stigmatized or discriminated against often try to hide their diagnosis from their colleagues, employers, and even their occupational physician [13,22,32]. Some PwMS report negative employer attitudes after disclosing the diagnosis. In addition, stigma, discrimination, and disclosure of MS in the workplace are potentially associated with an increased risk leaving the workforce prematurely [12,23]. People with disabilities who do not disclose may be deprived of accommodations that ensure job tenure [38]. Moreover, anticipated stigma and perceived discrimination were reported to discourage people with disabilities from pursuing employment or maintaining it [39]. It is also reasonable to imagine that stigma and discrimination may explain decisions of employment termination taken by the employer “based” on fictitious or misleading arguments. In this context, developing methods to counteract discrimination is of paramount importance in preventing unemployment of PwMS. Promoting the dissemination of accurate information in the workplace and demystifying misinformation can be a really effective strategy that doctors can lead.

The studies included in our review show that communication and information are key elements directly associated with the psychosocial characteristics of the work environment. Discrimination is mostly attributed to misinformation or a lack of information about the disease. In this context, the disclosure of the diagnosis of MS plays an important role in addressing the stigma and discrimination perceived by PwMS in the workplace. Changes in the psychosocial health of the working environment depend on an accurate understanding of the employees’ requirements. It is expected that health professionals should be more knowledgeable about MS and the PwMS who rely on their knowledge. Ironically, there is a significant proportion of workers that do not report the diagnosis of MS to their occupational physician. Because occupational physicians are often unaware of the diagnosis of MS, simple and cost-effective strategies based on the promotion of reliable information about MS in the workplace are undermined. Failure to communicate the diagnosis limits the occupational physician’s full potential to promote the occupational health of PwMS. Furthermore, management of debilitating symptoms can be optimized through early and supported disclosure of the illness [12,35]. 

There is a lack of experimental or quasi-experimental studies testing interventions to address workplace discrimination experienced by PwMS and promote trust for disclosure, although there is significant evidence in the literature of the benefits of these strategies on occupational outcomes in people with other diseases. A systematic review that involved 3854 study subjects demonstrated that anti-stigma interventions may be associated with improved employee knowledge and supportive behavior towards people with neuropsychiatric diseases [38]. McGahey et al. report that workers who completed a plan to manage their personal information that included the disclosure of the diagnosis had 4.9 times greater odds of employment at 6 weeks than those who preferred not to disclose any personal information [40]. One study included in our review listed the legal status of subjects with disabilities as an important predictor of disclosure, which is in line with previous similar findings for other disabling diseases [33,41,42].

To the best of our knowledge, this is the first systematic review addressing the stigma, discrimination, and disclosure of MS in the workplace. It provides the highest degree of evidence on this subject. This is also the first systematic review addressing these topics related to a non-communicable neurological disease. We performed searches in four different databases using a broad search strategy to reduce the chances that no relevant studies would be excluded. Most of the included studies have a high methodological quality, which minimizes the possibility of bias. Moreover, there was a large diversity of study designs, which strengthens the quality of evidence. This systematic review has also some limitations that need to be acknowledged to better interpret the results. Some studies used subjective and self-reported measures of stigma and discrimination which may introduce bias to the present results. The lack of objective measures may leave the interpretation of the results more complex and vulnerable to subjective speculations. We did not include data from the grey literature. Instead, we wanted to ensure that the data came from the scientific literature, have been peer-reviewed, and were as accurate as possible. We decided not to include specific terms in our search strategy due to the expected diversity in concepts and terms related to the central argument of the study and the inherent subjectivity of the outcomes. Lastly, we could not perform a meta-analysis due to the lack of similar types of data that could be pooled in a quantitative analysis. 

## 5. Conclusions

Stigma and discriminatory experiences were extensive in the context of work relationships among individuals with MS. Both feelings were closely associated with the disclosure of MS in the workplace. Strategies to combat MS-related stigma and discrimination in the workplace need to be investigated in future studies. The occupational physician is a central figure in promoting and ensuring the sustainable employability of PwMS.

## Figures and Tables

**Table 1:** Detailed search strategy in PubMed/MEDLINE, Scopus, SciVerse ScienceDirect, and Web of Science.

**PubMed**	(Employ* OR unemploy* OR occupation* OR “work” OR vocation* OR “workplace” OR “workforce” OR “labour force” OR “labor force” OR Career* OR Job* OR retire* OR “disability pension” OR “worker” OR “fitness for work”) AND (“Multiple sclerosis” OR “Demyelinating Autoimmune Diseases” OR “Demyelinating Autoimmune Disorders” OR “Clinically Isolated Syndrome” OR “Demyelinating”)
**Scopus**	TITLE-ABS KEY [(employ* OR unemploy* OR occupation* OR “work” OR vocation* OR “workplace” OR “workforce” OR “labour force” OR “labor force” OR career* OR job* OR “job retention” OR retire* OR “disability pension” OR “worker” OR “fitness for work”) AND (“Multiple sclerosis” OR “Demyelinating Autoimmune Diseases” OR “Demyelinating Autoimmune Disorders” OR “Clinically Isolated Syndrome” OR “Demyelinating”)]
**SciVerse Science Direct**	(“Employ” OR “occupation” OR “work” OR “vocation” OR “labour” OR “Job” OR “retire” OR “disability pension”) AND “Multiple sclerosis”
**Web of Science**	(Employ* OR unemploy* OR occupation* OR “work” OR vocation* OR “workplace” OR “workforce” OR “labour force” OR “labor force” OR Career* OR Job* OR retire* OR “disability pension” OR “worker” OR “fitness for work”) AND (“Multiple sclerosis” OR “Demyelinating Autoimmune Diseases” OR “Demyelinating Autoimmune Disorders” OR “Clinically Isolated Syndrome” OR “Demyelinating”)

**Table 2:** Description of the main characteristics of the studies included in the systematic review.

Authors	Year	Study Design	Country	N	Mean Age (SD)	Female Sex (%)	Mean Disease Duration (SD)	Progressive MS (%)	Main Results
Abbas et al. [13]	2008	Cross-sectional	France	76	41.5 (2.8)	59.0	9.0 (2.6)	21.0	Fifty-nine percent of the employers, 60% of the co-workers and 58% of the occupational physicians were aware of the existence of MS.
Abolhassani et al. [14]	2014	Qualitative	Iran	18	33.6 (7.1)	77.8	8.0 (5.2)	27.8	Participants noted that as soon as they announced the name of their illness, they would confront employment problems. Most of them also preferred to conceal their illness in their working environment due to their fear of losing their jobs.
Bass et al. [15]	2020	Cross-sectional	USA/ Germany/ Australia/ Canada/ France/ Italy/ Spain/UK	1075	31.2 (10.1)	NA	9.9 (7.1)	0.0	More than half of all respondents (68.3%) reported that most of their MS symptoms are hidden and that most people do not know that they have MS.
Benedict et al. [16]	2013	Cross-sectional	USA	52	44.8 (12.1)	NA	8.8 (7.7)	5.8	The majority (76.9%) reported having disclosed to their employer having MS, and there were no differences between disclosing and non-disclosing subgroups on any clinical characteristic.
Dorstyn et al. [17]	2017	Experimental	Australia	18	44.4 (9.2)	93.1	8.5 (7.7)	13.8	One in ten participants living with mild to moderate symptoms due to a relapsing-remitting subtype chose not to disclose their illness to previous employers to avoid the possibility of work conflicts.
Dorstyn et al. [18]	2018	Quasi-experimental	Australia	95	41.3 (9.8)	85.0	6.4 (7.4)	7.0	Participants who accessed a job information resource reported improved expectations in relation to the effect of MS symptoms on general self-esteem and work relations.
Fantoni-Quinton et al. [19]	2016	Cross-sectional	France	941	NA	79.8	NA	NA	Less than half of the respondents (48.4%) with an occupational activity after diagnosis stated that they spoke of their disease spontaneously before the presence of symptoms requiring a disclosure of their condition. The respondents stated that at some time, their health status was disclosed to their hierarchy (87.4% of respondents), colleagues (87.0%), occupational medicine physician (89.5%), and director of human resources (74.3%).
Frndak et al. [20]	2015	Cross-sectional	USA	199	45.8 (10.7)	18.9	9.4 (8.2)	7.0	There are three primary reasons for not disclosing at baseline: continued positive performance, fear of discrimination, new-hire status. Disclosure was associated with having worked longer for current employer, working more hours per week and using more accommodations.
Gill et al. [21]	2021	Qualitative	Ireland	6	NA	50.0	9.4 (NA)	NA	Some of the participants felt an obligation to disclose their diagnosis. It was a decision that they felt they had to make, and an essential part of keeping their job. Participants emphasised the importance of open communication in the initial stages of their diagnosis and how open communication continues as their employment role progresses.
Gregory et al. [22]	1993	Cross-sectional	New Zealand	80	NA	68.8	NA	NA	Just under 30% of the respondents did not inform their employers of them having MS. Most either did not want to draw attention to themselves or did not believe that MS affected their work at all. Several said that employers did not really understand the implications and that it would only become an issue if the employee proved to be functioning poorly in the job.
Hategeka et al. [23]	2019	Cross-sectional	Canada	530	50.7 (1.7)	74.9	18.9 (1.3)	NA	People with MS who reported greater feelings of stigmatization were more likely to be unable to work due to their MS, be cognitively impaired, less mobile and have a poorer quality of life.
Honan et al. [24]	2014	Cross-sectional	Australia	189	NA	NA	NA	NA	People with MS considered having a low self-esteem and perceiving their manager being not supportive of their condition as important work difficulties.
Jaworski et al. [25]	2021	Cohort	USA	70	43.3 (10.5)	67.1	10.4 (7.3)	4.3	A total of 46 (88.5%) PwMS still working at follow-up disclosed their MS status at work.
Kalantari et al. [26]	2018	Cross-sectional	Iran	305	32.0 (9.1)	74.8	7.4 (5.7)	NA	The average frequency of stigma in housewives and unemployed was higher than for other occupational groups and 43.8% of people with MS preferred not to mention their disease in job interviews.
Kirk-Brown et al. [12]	2014	Cohort	Australia	1438	44.7 (9.2)	83.0	NA	NA	Respondents with a more severe disability were more likely to disclose. Respondents who disclosed their MS to an employer were more likely to remain in employment over a three-year period and the odds of a person with MS remaining in employment increased by 1.30.
Kordovski et al. [27]	2015	Cross-sectional	USA	138	44.7 (10.0)	77.5	9.1 (7.3)	7.3	Eighty-two percent of patients reported disclosing disease status to their employer.
Krause et al. [28]	2021	Cross-sectional	USA	1234	48.0 (10.4)	77.6	NA	NA	One-third of people with MS believed that they would be discriminated against and would not be hired due to their disability.
Larocca et al. [29]	1996	Quasi-experimental	USA	43	41.6 (9.6)	75.6	7.5 (6.3)	NA	More than 90% disclosed MS to employers.
Maurino et al. [6]	2020	Cross-sectional	Spain	199	43.9 (10.5)	60.8	9.6 (7.1)	13.6	Perceived stigma was higher in unemployed than employed patients. Patients with Progressive MS and increased disability had increased perceived stigma.
Ongagna et al. [30]	2015	Cross-sectional	France	207	42.9 (8.7)	69.1	12.8 (6.1)	32.4	Seventy-seven per cent of the patients stated that their ‘professional entourage’ was aware of the diagnosis of their disease and 81.2% that the occupational physician was also aware.
Pérez-Miralles et al. [31]	2021	Cohort	Spain	55	47.3 (10.0)	43.6	NA	42.8	More than three-quarters reported some degree of stigma. Stigma was associated with a higher risk of depression and worse cognitive outcomes.
Reed et al. [32]	2017	Qualitative	USA	74	NA	NA	NA	NA	Several participants felt they were treated differently after disclosure. Fear of being discriminated against was also cited as a reason for not disclosing to future employers by several participants. Other participants delayed disclosure until after hiring and then if they decided to disclose, did so at the appropriate time for them, be it when their symptoms worsened, when they needed accommodations, or for other reasons.
Roessler et al. [33]	2016	Cross-sectional	USA	206	48.7 (12.7)	75.7	NA	45.6	Understanding the risks and benefits of disclosing disability status to employers was a concern for 97.2% of people with MS.
Rumrill et al. [34]	2015	Cross-sectional	USA	1924	54.0 (12.2)	78.7	NA	35.3	Knowing what to do if they encounter discrimination at work and understanding the benefits of disclosing disability status to employers were a concern for 96.5% and 96.4% of people with MS, respectively.
Rzepinski et al. [32]	2021	Cohort	Poland	375	43.1 (12.5)	69.3	NA	42.9	Informing the employer about the disease was associated with duration of patients’ professional activity.
Sweetland et al. [35]	2007	Cross-sectional	UK	24	NA	71.0	NA	21.0	Disclosure was seen by all the participants as a high risk strategy requiring considerable courage. Participants felt that support with disclosure was a significant priority for a specialist work service. It was felt that discrimination primarily resulted from lack of knowledge about MS. They also felt they would be more empowered to disclose at work if they better understood how they were protected legally and what was expected from their employers in terms of supporting them.

Note. SD: standard deviation, NA: not applicable.

**Table 3:** Quality appraisal of the studies included in the review.

Authors	Q1	Q2	Q3	Q4	Q5	Q6	Q7	Q8	Q9	Q10	Q11	Q12	Q13
Abbas et al. [13]	Yes	Yes	Yes	Yes	Yes	Yes	Yes	Yes					
Abolhassani et al. [14]	Yes	Yes	Yes	Yes	Yes	Yes	No	Yes	Yes	Yes			
Bass et al. [15]	Yes	Yes	Yes	Yes	No	No	Yes	Yes					
Benedict et al. [16]	Yes	Yes	Yes	Yes	No	No	Yes	Yes					
Dorstyn et al. [17]	Yes	No	Yes	No	No	No	Yes	Yes	Yes	Yes	Yes	Yes	Yes
Dorstyn et al. [18]	Yes	Yes	Yes	No	No	Yes	Yes	Yes	Yes				
Fantoni-Quinton et al. [19]	No	No	Yes	Yes	Yes	No	Yes	Yes					
Frndak et al. [20]	Yes	Yes	Yes	Yes	Yes	Yes	Yes	Yes					
Gill et al. [21]	Yes	Yes	Yes	Yes	Yes	Yes	Yes	Yes	Yes	Yes			
Gregory et al. [22]	No	No	No	No	No	No	No	No					
Hategeka et al. [23]	No	Yes	Yes	Yes	Yes	Yes	Yes	Yes					
Honan et al. [24]	Yes	Yes	Yes	Yes	Yes	Yes	Yes	Yes					
Jaworski et al. [25]	Yes	Yes	Yes	Yes	No	Yes	Yes	Yes	Yes	No	Yes		
Kalantari et al. [26]	No	Yes	Yes	Yes	NA	NA	Yes	Yes					
Kirk-Brown et al. [12]	Yes	Yes	Yes	Yes	Yes	Yes	Yes	Yes	No	No	Yes		
Kordovski et al. [27]	Yes	Yes	Yes	Yes	No	No	Yes	Yes					
Krause et al. [28]	Yes	Yes	Yes	Yes	Yes	No	Yes	Yes					
Larocca et al. [29]	Yes	Yes	Yes	Yes	No	Yes	Yes	Yes	Yes				
Maurino et al. [6]	Yes	Yes	Yes	Yes	Yes	Yes	Yes	Yes					
Ongagna et al. [30]	Yes	Yes	Yes	Yes	NA	NA	Yes	Yes					
Pérez-Miralles et al. [31]	NA	NA	Yes	Yes	No	No	Yes	No	Yes	NA	Yes		
Roessler et al. [33]	No	Yes	Yes	Yes	NA	NA	Yes	Yes					
Rumrill et al. [34]	No	Yes	Yes	Yes	NA	NA	Yes	Yes					
Rzepinski et al. [36]	NA	NA	Yes	Yes	No	NA	Yes	Yes	NA	NA	Yes		
Sweetland et al. [35]	Yes	Yes	Yes	Yes	NA	NA	Yes	Yes					
Reed et al. [32]	Yes	Yes	Yes	Yes	Yes	Yes	No	Yes	Yes	Yes			

Note. NA: not available.

## Data Availability

Data can be found in Table 2.
